# Implications for Emotion: Using Anatomically Based Facial Coding to Compare Emoji Faces Across Platforms

**DOI:** 10.3389/fpsyg.2021.605928

**Published:** 2021-02-25

**Authors:** Jennifer M. B. Fugate, Courtny L. Franco

**Affiliations:** ^1^Department of Psychology, University of Massachusetts Dartmouth, Dartmouth, MA, United States; ^2^Department of Communication and Information Science, University of Alabama, Tuscaloosa, AL, United States

**Keywords:** emoji faces, emotion perception, facial action coding system, electronic platforms, facial expressions

## Abstract

Emoji faces, which are ubiquitous in our everyday communication, are thought to resemble human faces and aid emotional communication. Yet, few studies examine whether emojis are perceived as a particular emotion and whether that perception changes based on rendering differences across electronic platforms. The current paper draws upon emotion theory to evaluate whether emoji faces depict anatomical differences that are proposed to differentiate human depictions of emotion (hereafter, “facial expressions”). We modified the existing Facial Action Coding System (FACS) (Ekman and Rosenberg, 1997) to apply to emoji faces. An equivalent “emoji FACS” rubric allowed us to evaluate two important questions: First, *Anatomically, does the same emoji face “look” the same across platforms and versions?* Second, *Do emoji faces perceived as a particular emotion category resemble the proposed human facial expression for that emotion?* To answer these questions, we compared the anatomically based codes for 31 emoji faces across three platforms and two version updates. We then compared those codes to the proposed human facial expression prototype for the emotion perceived within the emoji face. Overall, emoji faces across platforms and versions were not anatomically equivalent. Moreover, the majority of emoji faces did not conform to human facial expressions for an emotion, although the basic anatomical codes were shared among human and emoji faces. Some emotion categories were better predicted by the assortment of anatomical codes than others, with some individual differences among platforms. We discuss theories of emotion that help explain how emoji faces are perceived as an emotion, even when anatomical differences are not always consistent or specific to an emotion.

## Implications for Emotion: Using Anatomically Based Facial Coding to Compare Emoji Faces Across Platforms

Emojis, which are now incorporated into people’s everyday channels of nonverbal communication, are assumed to represent, or at least resemble, human facial depictions of emotion (hereafter, referred to as “facial expressions”). Despite many studies that show that including emojis alters emotional content, only one study (Franco and Fugate, 2020) has examined whether emoji faces are perceived as a discrete emotion. A logical next step is to explore whether emoji faces are structurally “equivalent” among platforms and version updates, and whether emoji faces actually resemble prototypical facial expressions (in physical appearance).

In this paper, we adapted the Facial Action Coding System (FACS) (Ekman and Rosenberg, 1997) to systematically compare emoji faces with respect to facial movements, called action units (AUs). Although AUs are built on changes in the underlying facial musculature, movements can be inferred in still faces based on deviations from a baseline pose. We used this adaptive new system to code 31 emojis on their physical appearance across two different versions of three electronic platform carriers (Apple iOS 9.1, Apple iOS 13.3, Google Android 6.0, Google Android 10, Samsung TouchWiz 5.1 and Samsung One UI 1.5). We then systematically compared the AUs within and between emojis across platforms and versions. We also used previously collected data of participants’ emotion category assignment for each emoji (Franco and Fugate, 2020) to see whether the emoji AUs conformed to those proposed for human facial expressions, according to the literature (Cordaro et al., 2018).

### A Brief Primer on Emotion Theory and “Basic” Facial Expressions

According to some theories of emotion, facial expressions dissociate themselves reliably among emotions (Tomkins, 1962; Izard, 1991, 1992, 2013; Ekman, 1992, 2016; Matsumoto et al., 2008; Brosch et al., 2010; Sauter et al., 2011; for an extensive review see Barrett et al., 2019). Furthermore, under this view, a “basic” set of emotions are viewed as innate and universal among individuals (Ekman and Cordaro, 2011). According to these views, emotions are also biologically based and evolutionary-preserved, such that facial expressions have evolutionary significance and are shared with taxonomically related species (van Hooff, 1962; Ekman, 1972, 1992; Matsumoto, 1989; deWaal, 2003; Parr et al., 2007). This view comes mainly from similar mimetic facial musculature that is highly conserved across primates (Huber, 1931; Parr et al., 2007). The human and non-human mimetic facial musculature have been anatomically mapped by a system of action units that are shared between species (e.g., chimpFACS: Parr et al., 2007; and MaqFACS: Parr et al., 2010). Although the early work on emotion perception and facial musculature focused on six “basic” emotions (e.g., anger, disgust, sadness, happiness, fear, and surprise) (Ekman, 1972, 1992; Ekman et al., 1983; for reviews, see Elfenbein et al., 2002; Keltner et al., 2016, 2019), more recent research has proposed more than twenty “basic” emotions might exist based on self-report (Cowen and Keltner, 2017). Fourteen of these emotions show at least some consistency in the AUs identified for the emotion prototype across multiple studies (see Table 1). In our initial research (Franco and Fugate, 2020), we explored the nine most common emotions (plus envy).

**TABLE 1:** AU Prototypes across Literature.

	Ekman et al., 1983	Keltner and Buswell, 1997	Shiota et al., 2003	Matsumoto et al., 2008	Du et al., 2014	Keltner and Cordaro, 2015	Cordaro et al., 2016	Cordaro et al., 2018 (ICP reported)
Amusement			6,12,26 or 27, 55 or 56*	−	−	6,7,12, 25,26,53		6,7,12, 16,25,53*
Anger ∧	4,5,7,23			4,5 or 7, 22, 23,24	4,7,(10),(17),(23), 24	4,5,17, 23,24		4,7
Awe			1,5,26 or 27,57*	−	1,2,(4),5,(20),25, (26)	−		1,2,5,12,25,53*
Contempt∧	12,14			12,14				4,14,25
Contentment∧						12,43	12,43	12,43*
Sex/Desire (Love) ∧						19,25,26,43		6,7,12,25
Disgust∧	9,15,16			9 or 10, (25 or 26)	(4),9,10,17, (24)	7,9,19, 25,26		4,6,7,9, 10,25,26*
Embarrassment		12,24,51, 54,64		−	−	7,12,15,52,54,64		6,7,12, 25,54*
Fear∧	1,2,4,5,7, 20,26			1,2,4,5, 20, (25 or 26)	1, (2),4,(5),20,25, (26)	1,2,4,5,7,20,25		1,2,5,7, 25*
Happiness∧	6,12			6,12	(6),12,25	6,7,12, 25,26		6,7,12, 16,25,26*
Pride			6,12,24,53*	−	−	53,64		7,12,53*
Sadness∧	1,4,15^1^			1, (4),15, (17)	(1),4, (6),(11),15,(17)	1,4,6,15,17		4,43,54
Shame		54,64		−	−	54,64		4,17,54
Surprise∧	1,2,5,26			1,2,5,25 or 26	1,2,(5), 25,26	1,2,5,25,26		1,2,5,25

We compared our codes to the last column. * = additional head or posture movement indicated, but no AUs identified for such. ∧ = used in the current paper. Envy is also used, but there are no proposed AUs for this emotion. ^1^The original paper listed the “15” as a “5”. We believe this to be a mistake and therefore corrected it.

Cordaro et al. (2018) collected free-response facial and bodily responses to emotional statements from Chinese, Indian, Japanese, Korean, and American individuals. Of the emotions investigated in the current paper, surprise, contentment, fear, and anger all had over 50% overlap with the proposed emotional prototype based solely on AUs (89%, 80%, 71%, 67%, respectively), whereas contempt, sadness, and disgust showed less than 50% overlap with the proposed emotional prototype (33%, 33%, 18%, respectively). From these similarities and differences, they concluded that approximately 50% of an individual’s overall expressed facial movements represent the universal prototype, whereas another 25% are due to the culture’s “emotional dialect.” Some of these structural changes in the facial musculature are known to be less diagnostic (e.g., wide eyes) and are shared among emotion categories (e.g., fear and surprise) (see Keltner et al., 2019; for an excellent review of human facial “expressions” and emotion, see Barrett et al., 2019). Despite such cultural variations, many of these researchers still continue to accept the universality of human facial expressions (hence the term “expressions” rather than facial “movements”) and have introduced the International Core Pattern (ICP) of AUs for each of the emotions (see Table 1).

Other researchers highlight the cultural variation of facial expressions while still prescribing to a correspondence between facial expressions and emotion. For example, Elfenbein et al. (2007) and Elfenbein (2013) coined the “dialect theory of emotional expression,” which posits that emotional expressions have regional or linguistic dialects (Elfenbein et al., 2007).

Other theories of emotion treat emotions as *products* of a person’s brain to categorize more generalized affective information, which alone is not diagnostic of a particular emotion category. For instance, the Theory of Constructed Emotion (formerly known as Psychological Constructionism) posits that emotions are constructed through a person’s conceptual knowledge within a given context (Barrett, 2006a,b, 2017). According to this theory, there are likely to be no distinctive or prescriptive emotional indicators for a specific emotion in the face (e.g., AUs). Even though frowns and smiles provide differences in structural information, perceivers must learn to associate them with sadness and happiness. Such associations are learned when another person labels the face with an emotion word (e.g., sad or happy), or a person uses situational knowledge to contextualize the information (Betz et al., 2019; for reviews, see Lindquist et al., 2016; Barrett, 2017; Lindquist, 2017). Therefore, a person’s conceptual knowledge and context play a large role in the formation of facial depiction-emotion associations, and by extension, would likely also contribute to the perception of emotion from emoji faces. These ideas are consistent with how people develop electronic communication skills, more generally. That is, people develop an understanding of what another means through experience with others and feedback on that information (Ling, 2010; Liu and Yang, 2016).

Considering that emoji faces were designed to convey emotional content and to (presumably) resemble human facial expressions, it is worth comparing whether software companies’ depictions actually capture the physical resemblance to certain facial expressions. Little scholarship provides insight to why multiple variations of the same emoji exist in the first place (Bailey, 2018; Lee, 2018). That is, there is little information on how individual emoji were “translated” across platforms, only that there is one translation through the Unicode system (Toratani and Hirayama, 2011; Unicode, 2020). Much like how facial expressions vary across cultures, emoji sets vary across platforms. Thus, the specific renderings of an emoji belong to web creators or web developers (e.g., Apple, Samsung, Google, Facebook, Twitter, etc.). Too much overlap between the “same” emoji on different platforms and/or version updates might be considered copyright infringement and could result in litigation (Bailey, 2018). While the Unicode has one “translation” for each emoji across platforms and version updates, it is up to developers and web creators to decide exactly *what* each translated emoji will look like.

Some researchers have alluded to the fact that emojis are artistic creations or creative expressions (Lee, 2018). To this end, emojis are considered art and not meant to be realistic depictions (of facial movements, in this case). As with any work of creative art, it is therefore up to the artist to communicate the intention even when the representation is not apparent^1^.

### Previous Literature of Emoji Emotional Perception

People perceive emoji faces similarly to human emotion faces. For example, Gantiva et al. (2019) found that emoji faces produced similar neural responses to real faces observed during face-to-face communication. In another study, Yuasa et al. (2011) found that emojis and human facial expressions elicited similar brain activity in the right and left inferior frontal gyri. Other areas within the brain, known to be important in processing emotional faces (e.g., right fusiform gyrus), were not significantly activated by emojis, however. A recent fMRI study investigated memory retrieval for emotional emoji faces and found significant activation within the inferior frontal gyrus, amygdala, and right temporal pole (Chatzichristos et al., 2020).

A growing body of research aims to understand how people use emojis to relay emotional sentiment on social media platforms, such as Twitter and Facebook. In 2015, researchers categorized 96,269,892 tweets by emotional content to find overarching patterns of emoji sentiment (reported in Wolny, 2016). For example, researchers categorized tweets containing grinning emoji faces as having positive sentiment. The study reduced approximately 90% of all emojis into just four emotion categories: happy, sad/unhappy, undecided/skeptical, and surprise/shock (Wolny, 2016). The results suggested that many different emojis can be used interchangeably to communicate an emotion. In a more recent and even larger study, Felbo et al. (2017) conducted a sentiment analysis on 1,246 million tweets containing one of 64 common emojis. They examined emoji occurrences to learn sentiment, emotion, and sarcasm. They found that emoji use was structured by a combination of linguistic and social contexts, as well as cultural convention.

Only a handful of empirical research has investigated the relationship between perceived emotion category and emoji faces, however (Oleszkiewicz et al., 2017; Betz et al., 2019; Franco and Fugate, 2020). Oleszkiewicz et al. (2017) asked children to view real human and emoji faces and identify the emotion. Children assigned human faces and emoji faces with high agreement to the categories “happy” and “sad,” yet there was only low agreement for the other basic emotions (e.g., fear, anger, surprise, and disgust) for both emoji and human faces. Another study (Betz et al., 2019) found that emotion words served as a context for perceiving emotions from the Finch faces, emoji-like faces created by Pixar illustrator, Matt Jones (Jones, 2017). This particular set of emoji faces was created based on Darwin’s depictions of basic emotional “expressions” in man and animals (Darwin, 1872/2005). Despite these faces being created to specifically exemplify emotional “expressions,” participants had overall low agreement about which emotion was displayed unless they were forced to choose from a provided emotion word. In our previous work, we found only about half of the emojis explored were assigned at statistically higher rates to one emotion category compared to another, and less than one sixth of the faces were specific to an emotion category (meaning that they were not also affiliated with another emotion at similar levels) (Franco and Fugate, 2020).

A handful of studies have shown that emoji rendering differences among electronic platforms may lead to miscommunication and misinterpretation (Miller et al., 2016; Tigwell and Flatla, 2016; Miller Hillberg et al., 2018; Rodrigues et al., 2018). Miller Hillberg et al. (2018) found that 25% of Twitter users were unaware that emojis’ appearances change depending on a user’s electronic platform. Additionally, 20% of users reported that they would edit their emoji selection or tweet after being shown rendering differences. And, in our previous research, we found that there were significant differences in what emotions people associated most intensely with an emoji face, depending on the electronic platform they viewed them on (Franco and Fugate, 2020).

In another study assessing electronic platform differences, users evaluated a randomized subset of 20 emoji faces on two platforms for their esthetic appeal, familiarity, visual complexity, concreteness, valence, arousal, and meaningfulness (Rodrigues et al., 2018). Users also provided a free response as to what they thought the emoji meant or what emotion they thought it represented. Although the individual free responses were not provided for each emoji in the article, overall agreement (after coding for similarities) of responses was slightly greater for iOS emojis (66.78%) than for the same emojis on Android (64.95%). Moreover, iOS ratings on “meaningfulness amount” were statistically higher for iOS (when bootstrapped) compared to those for Google Android.

### The Current Study

In this paper, we first adapted the Facial Action Coding System (FACS) to systematically compare emoji faces with respect to anatomical-based changes (AUs). We compared 31 emojis (spanning ten emotions) on their appearance across three electronic platform carriers, each with two different version updates (Apple iOS 9.1, Apple iOS 13.3, Google Android 6.0, Google Android 10.0, Samsung TouchWiz 5.1, and Samsung One UI 1.5) (see Table 2). The creation of a coding rubric for schematic faces is essential in order to compare anatomically and reliably the renderings of an emoji across platforms and versions. Therefore, our goal in creating such a rubric was to be able to use it to address two fundamental questions about the relationship between the physical renderings of emoji faces on different among platforms (and versions), and their relationship to human facial expressions.

**TABLE 2:** Emojis for both Apple Platform Versions with FACS Code and Perceived Emotion (Additional Platforms Below).

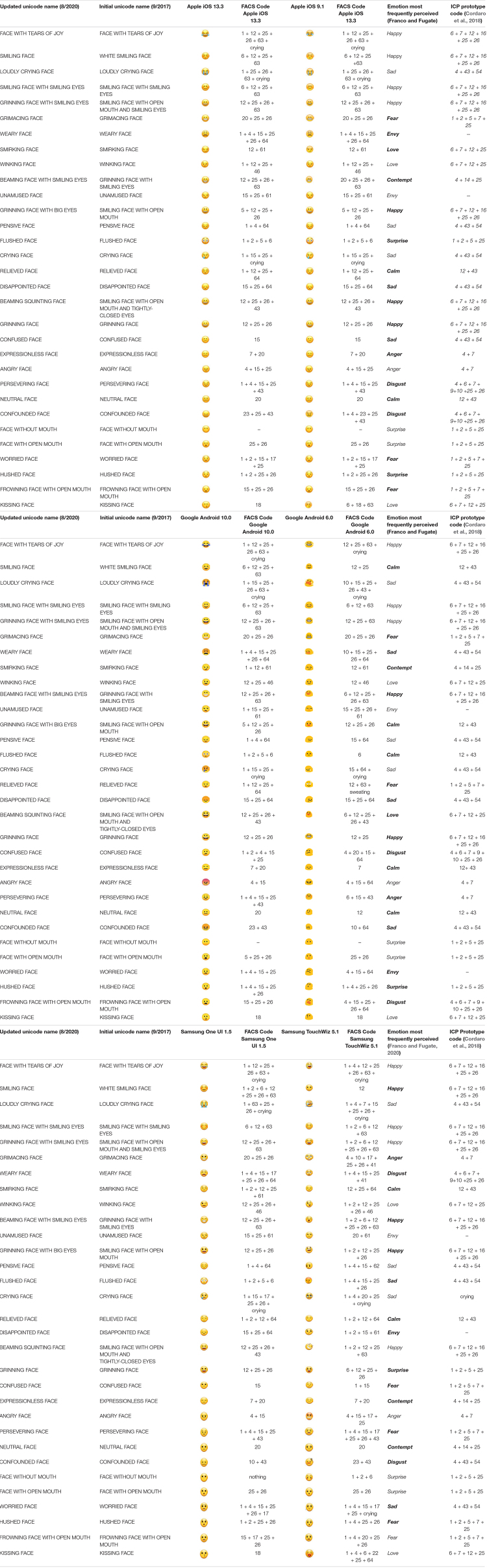

*Bolded emotions are those which were perceived as different across platforms.*

The first goal was to assess objectively *physical appearance*. Specifically, are the anatomical-based changes (AUs) of an emoji face the same across electronic platforms and version updates? Said another way: *Anatomically, does the same emoji face “look” the same across platforms and versions?* After using our adaptive emoji-FACS rubric to code each emoji face, we then systematically compared the distribution and frequencies of AUs across emoji faces by platforms and versions.

***Hypothesis 1a:*** Action units (as an objective measure of facial coding) should reflect the known perceptual differences users encounter when an emoji is sent from another platform. For the same set of emojis, AU counts and distributions should differ among platforms/versions.

***Hypothesis 1b:*** If emoji faces represent facial “expressions,” those faces perceived as the same emotion across platforms should be more similar in AU counts and distributions compared to those which are perceived as different emotions.

The second goal was to assess *emotional meaning*. Specifically, do the anatomical-based changes of an emoji face reflect those proposed for human facial depictions of emotion? Said another way: *Do emoji faces perceived as an emotion category resemble human facial depictions of the same emotion category?* To assess this goal, we compared the AUs we coded for emoji faces to the the prototypical AUs (ICPs) described for facial expressions (according to the literature, see Cordaro et al., 2018).

***Hypothesis 2a:*** If emoji faces resemble human facial “expressions,” then there should be a high correspondence among AUs for an emoji face (perceived as an emotion) and the human facial depiction for that emotion.

***Hypothesis 2b:*** If emoji faces resemble human facial “expressions,” then the AUs should significantly predict (classify) the perceived emotion category.

## Methods

### Stimuli Sets of Emojis

We began by using the 31 emojis from Apple iOS 9.1 (hereafter called Apple 9.1), Google Android 6.0, and Samsung TouchWiz 5.1 (hereafter called Samsung Wiz) that were identified as belonging to ten different emotion categories in Franco and Fugate (2020). The emotions investigated in that paper were ten of those listed as being basic emotions, and included anger, calm (called contentment according to Cordaro et al., 2018), contempt, fear, envy^2^, disgust, happiness, love (called sex/desire according to Cordaro et al., 2018), sadness, and surprise. All emojis were represented in the Unicode Standard System (see Table 2 for Unicode name). We then added the equivalent, most up-to-date (at the time this project began) emojis from each of these platforms (also listed in Table 2). Therefore, we used 31 emojis, which were represented on each of two versions for the three platforms (e.g., Apple 9.1, Apple iOS 13.3 (hereafter Apple 13.3); Google Android 6.0, Google Android 10.0; and Samsung Wiz, Samsung One UI 1.5 (hereafter Samsung One) (Emojipedia, 2020). All emoji face names are referred to by the newer, updated Unicode name.

### Coding of AUs

Both coders were certified FACS-coders, with over 25 years of combined experience, who completed their training with Erika Rosenberg and used the FACS Investigator Guide to code^3^.

The first author set some initial guidelines as to what was considered “baseline” for schematic faces. The initial guidelines included the following marks as “baseline”: (1) circle eyes, as long as not oval or extra-large; (2) straight line mouths, as long as not elongated; (3) straight line eyebrows (when present; not all emojis have eyebrows and marks were only considered eyebrows if there was also an eye). The second coder agreed to these assumptions. Both coders agreed to not code intensities of AUs or to code head movements or miscellaneous codes^4^. Both coders initially coded unilateral movements, but later dropped right and left designations in the final codes for simplicity^5^.

Both coders independently came up with a list of AUs that they could conceivably code. This included 25 AUs (in chronological order): 1 (inner brow raise), 2 (outer brow raise), 4 (brow lowerer), 5 (upper lid raise), 6 (cheek raiser)^6^, 7 (lid tightener), 10 (upper lip raiser), 12 (lip corner puller), 14 (dimpler), 15 (lip corner depressor), 16 (lower lip depressor), 17 (chin raiser), 18 (lip pucker), 20 (lip stretch), 22 (lip funneler), 23 (lip tightener), 25 (lip part), 26 (jaw drop), 41 (lid droop), 43 (eyes closed), 46 (wink), 61 (eyes left), 62 (eyes right), 63 (eyes up), and 64 (eyes down).

Both coders then produced a depiction(s) of each AU and sent it to one another. Together, they combined different variations for each AU. There was some initial debate over AU 10, 23, and 22. Renderings for all three of these AUs were agreed upon after discussion (see Table 3 for final depictions). After discussion, the two coders came to agreement through conversation, and eventually both used Table 3 as the final coding rubric.

**TABLE 3:** Coding Rubric.

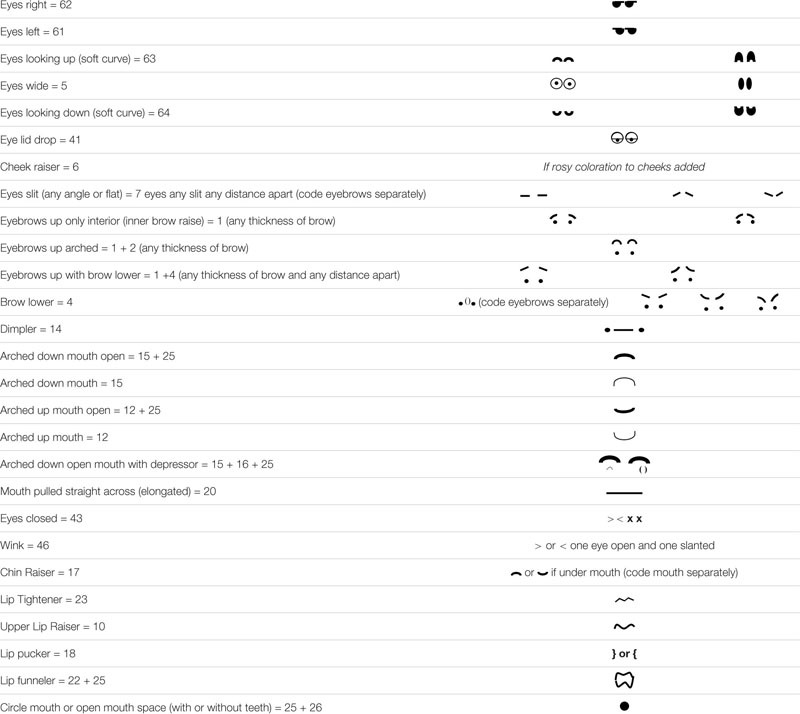

*The coders looked through the basic set of AU codes (excluding miscellaneous and head positions) to see which AUs they both could conceivably imagine what that AU might look like in a schematic form. This included 25 AUs (in chronological order): 1, 2, 4, 5, 6, 7, 10, 12, 14 (none noted), 15, 16 (none noted), 17, 18, 20, 22, 23, 25, 26, 41, 43, 46, 61, 62, 63, 64. Of the AUs indicated for the ICP emotion prototypes for the nine (note envy does not have a code) emotions investigated, only AU 9 did not have a code, and AU 54 was not included because no head positions were included. AUs in which neither coded could conceive of what it might look like, were not included: AU 9 (nose wrinkle; no noses in emojis), 13 (sharp lip puller), 24 (lip press, unable to distinguish might be how different from 23), 27 (mouth stretch), 28 (lip suck), 45 (blink), 65 (walleye), and 66 (Crosseye). “Absence” codes were not used [AU 70,71 (brows and eyes not visible, respectively), and 72 (lower face not visible)]. Circle eyes are considered “normal,” whereas straight line (un-elongated) mouths are considered “normal.” Eyebrows only assumed if eye also present. Straight line eyebrows are considered normal. Straight line mouth (not elongated) is considered “normal.” Head positions and miscellaneous codes not included.*

AUs in which neither coder could conceive of what it might look like were not included in the rubric. These included: AU 9 (nose wrinkle; no noses in emojis), 13 (sharp lip puller; unable to distinguish from AU 12 or 14), 24 (lip press; unable to distinguish from AU 23), 27 (mouth stretch; unable to distinguish between open mouth, AU 25 and AU 26), 28 (lip suck; unable to imagine), 45 (blink; no movement), 65 (walleye; unable to imagine), and 66 (crosseye; unable to imagine). “Absence” codes were not used (AU 70, 71) (brows and eyes not visible, respectively), and 72 (lower face not visible). Of the AUs indicated for the ICP emotion prototypes for the nine emotions investigated, only AU 9 and AU 54 were not included in the rubric.

Finally, both coders noted that there were two additional “embellishments” that were seen regularly on emoji faces and might be important to code: this include a tear (which was called the *crying code)* and a “tear” but alongside the upper face (not eye) (which was called the *sweating code)*. Although there is no AU for crying or sweating in FACS, tears and sweat have been proposed as possible emotional outputs.

### Reliability

Each coder first coded ten random emoji faces (from different platforms and versions). The first coder compared the AUs between her and the other coder. Reliability was greater than 89%, and the coders resolved any disagreements, which resulted in 100% agreement on the final code for the first ten emojis in the file.

Each coder then used the rubric to code the rest of the emoji faces, which were presented randomly by platform, one emoji per page in a file. There were two files total, which divided the earlier version from the later version. The first author then calculated the reliability for each emoji face, for each platform, for each version. Reliability was calculated by scoring a “1” for any AUs indicated by only one coder and a “2” for any AUs agreed upon by both coders. The total number of AUs counted was then added. Finally, the summed count of AUs from the coders was divided by the AUs counted multiplied by two. The overall reliability between coders on Apple 9.1 was 75% (ranging from 67 to 100% across faces, *n* = 9 faces had perfect agreement); on Apple 13.3, 94% (ranging from 70% to 100%, *n* = 19 had perfect agreement); on Google Android 6.0, 88% (ranging from 50% to 100%, *n* = 12 had perfect agreement); on Google Android 10.0, 96% (ranging from 83% to 100%, *n* = 22 had perfect agreement); on Samsung Wiz, 88% (ranging from 67% to 100%, *n* = 10 had perfect agreement); and finally on Samsung One, 93% (ranging from 63 to 100%, *n* = 20 had perfect agreement). The overall reliability between the coders across platforms and versions was 91%. In cases in which the codes did not match, the first author made the final decision and included it as the “final code” in Table 2. The second coder approved the final codes.

## Results

### Overall Use of AUs

Twenty seven coded AUs (including *crying* and *sweating*) were identified on the coding rubric. Table 4 shows the percentage of time each AU was coded across all platforms/versions. AU 14 and AU 16 were never coded in any emoji face. Statistical significance was conducted with an alpha of .05 two-tailed, unless indicated otherwise.

**TABLE 4:** AU counts by Platform/Versions.

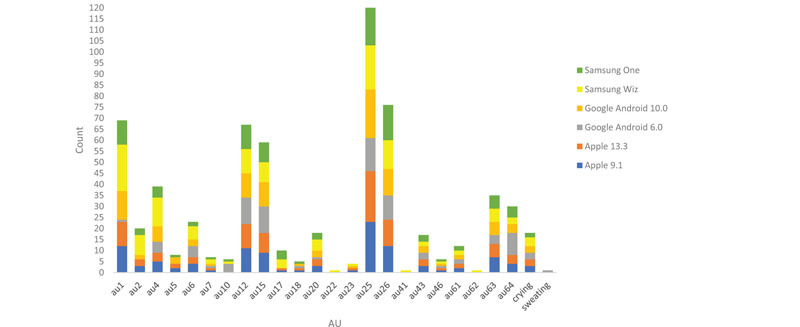

### Analysis 1a: Counts and Distribution of AUs

#### Across All Platforms and Versions

Both Apple 9.1 and 13.3 used 20 of the 25 AUs across emoji faces. Both platforms did not use AUs 10, 22, 41, 62, or the sweating code (Table 4).

Google Android 6.0. used 18 AUs, and Google Android 10.0 version used 19 AUs. Neither version used AU 17, AU 22, AU 41, or AU 62. Google Android 6.0 also did not use AU2, AU 5, or AU 23, whereas Google Android 10.0 also did not use AU10 or *sweating.*

Samsung Wiz used 22 AUs and Samsung One used 20 AUs. Neither used *sweating.* Samsung Wiz did not use AU5 and AU 18, whereas Samsung One did not use AU 22, AU 23, AU 41, and AU 62.

To assess Hypothesis 1a overall, we compared the overall AU count from the 25 AUs for which we had data across the three platforms and versions.

Because our data was not normally distributed, we used a Kruskal-Wallis test for both the overall AU count and AU distribution. For the overall AU count, there was a significant difference among the platforms/versions, *H*(5) = 11.844, *p* < 0.05 (Mean rank Apple 9.1 = 93.76; Apple 13.3 = 89.27; Google Android 6.0 = 74.68; Google Android 10.0 = 94.42; Samsung Wiz = 119.50; Samsung One = 89.37). When controlling for multiple comparisons (Bonferroni), only Google Android 6.0 and Samsung Wiz differed statistically from each other, *U*(2) = −44.823, *p* < 0.05.

To test differences in the distribution of individual AUs, we again performed a Kruskal-Wallis test on each AU. Three AUs differed statistically across platforms/versions. The first was AU 1, *H*(5) = 27.980, *p* < 0.05, in which Google Android 10.0 and Samsung Wiz differed statistically (controlling for multiple comparisons) (*p* < 0.05). AU 2 also differed, *H*(5) = 15.157, *p* < 0.05, in which Google Android 6.0 and Samsung Wiz differed (controlling for multiple comparisons) (*p* < 0.05). AU 10 also differed across platforms/versions, *H*(5) = 12.333, *p* < 0.05, but no follow-up comparisons remained significant after controlling for multiple comparisons. The distribution of AU 17 was marginally significant across platforms/versions, *H*(5) = 10.932, *p* = 0.053.

#### Individual Platforms

We next investigated whether the AUs differed between the older and newer versions of emojis for each platform.

#### Apple Versions

Between the two versions of Apple (9.1 and 13.3), there were very few obvious physical differences between the corresponding emojis. The one exception was the original “beaming face with smiling eyes,” which was replaced with the “grinning face with smiling eyes,” and was physically quite different (see Table 2).

There was no difference between the Apple versions on overall count of AUs, *U*(2) = 455.5, *p* > 0.05. None of the individual AUs between Apple versions were significant either.

### Android Versions

The majority of emojis between the two versions of Google Android (6.0. and 10.0) were noticeably different from just looking at them. Most apparent was that the gum-drop shaped head of the original version was replaced with the more standard circle head. Thus, version 10.0 appeared more similar to the other platforms. In addition, the newer version used the yellow-orange color variation of faces seen in the other platforms and versions. The large red “blob mouths” were replaced with lines, again converging with the other platforms and versions.

The difference between the overall AU count, however, was not statistically significant, *U*(2) = 591.00, *p* > 0.05. AU 1 and AU 10 differed significantly between the two versions, *U*(2) = 666.50 and *U*(2) = 418.5, *ps* < 0.05.

### Samsung Versions

About half of the emojis looked noticeably different between the two versions of Samsung (Wiz and One) (see Table 2). Specifically, the newer version had emojis looking straight on, whereas the older version had several emojis with head turns and tilts.

The difference between the overall AU count was statistically significant, *U*(5) = 336.00, *p* < 0.05. AU 1 and AU 4 differed between the two versions, *U*(2) = 325.50 and *U*(2) = 356.5, *ps* < 0.05. AU 2 was marginally significant, *U*(2) = 387.5, *p* = 0.056.

### Summary

With respect to Hypothesis 1a, some platforms and versions had different total AU counts and different distributions of AUs. Overall, Google Android 6.0 had the fewest countable AUs (*n* = 19), even though it only had only slightly fewer AUs than most of the other platforms.

Between the two versions of Apple, there was no difference in the overall AU count or distribution of AUs overall. Between the two versions of Google Android, although there were no significant differences across overall AU count, there were differences in the counts for two individual AUs. Finally, there was a significant difference for both overall AU count between versions of Samsung, and three individual AUs differed statistically (or marginally so).

For a more detailed look of AU correlations between versions/platforms by individual emoji face, we refer the reader to Table 5 which lists the correlation coefficients (based on Spearman’s rho).

**TABLE 5:** Correlation Coefficients for each Emoji Face among all AUs by Platforms and Versions.

**Unicode Name (8/2020)**	**Apple 9.1 and Apple 13.3**	**Google Android 6.0 and Google Android 10.0**	**Samsung Wiz and Samsung One**	**Apple 9.1 and Google Android 6.0**	**Apple 9.1 and Samsung Wiz**	**Google Android 6.0 and Samsung Wiz**	**Faces Perceived as Same Emotion Across Platforms**	**Faces Perceived as Different Emotion Across Platforms**
	**Between versions of the same** **platform**			**Between older versions of each platform (for which perception data exists) (see right columns)**				
Angry Face	1.0	0.700	0.677	0.623	**0.874**	0.513	Angry	–
Beaming Face with Smiling Eyes	1.0	0.874	0.703	**0.874**	0.703	*0.804*	–	Contempt: A – Happy: G & S
Beaming Squinting Face	1.0	0.874	0.333	**0.874**	*0.333*	**0.257**	–	Happy: A & S – Love: G
Confounded Face	0.740	–0.803	0.458	–0.141	**0.592**	–0.083	–	Disgust: A– Sad: G & S
Confused Face	1.0	0.333	0.693	0.469	**0.693**	0.277	–	Sad: A – Disgust: G – Fear: S
Crying Face	1.0	0.513	0.196	0.513	**0.603**	0.129	Sad	–
Disappointed Face	1.0	1.0	0.180	***1.0***	0.180	0.180	–	Sad: A & G – Envy: S
Expressionless Face	1.0	0.693	1.0	0.693	**1.0**	0.693	–	Anger: A– Calm: G – Contempt: S
Face with Open Mouth	1.0	0.799	1.0	**1.0**	**1.0**	**1.0**	Surprise	–
Face with Tears of Joy	1.0	0.778	0.902	0.778	**0.902**	0.703	Happy	–
Face without Mouth (no AUs coded)	NA	NA	NA	NA	NA	NA	Surprise	–
Flushed Face	1.0	0.469	0.129	**0.469**	0.062	–0.098	–	Surprise: A – Calm: G – Sad: S
Frowning Face with Open Mouth	1.0	0.740	0.333	**0.740**	*0.435*	0.505	–	Fear: A & S– Disgust: G
Grimacing Face	1.0	1.0	0.374	***0.435***	0.374	0.428	–	Fear: A & G– Angry: S
Grinning Face	1.0	0.799	0.847	*0.799*	**0.847**	0.677	–	Happy: A & G– Surprise: S
Grinning Face with Big Eyes	1.0	0.847	0.740	**0.847**	*0.603*	0.740	–	Happy: A & S– Calm: G
Grinning Face with Smiling Eyes	1.0	0.833	0.677	**0.833**	0.677	0.778	Happy	–
Hushed Face	1.0	0.874	0.677	*0.705*	**1.0**	**1.0**	–	Surprise: A & G– Fear: S
Kissing Face	0.554	1.0	−0.110	**0.554**	0.088	–0.110	Love	–
Loudly Crying Face	1.0	0.513	0.196	0.428	0.584	0.491	Sad	–
Neutral Face	1.0	–0.040	1.0	*−0.040*	**1.0**	–0.040	–	Calm: A & G– Contempt: S
Pensive Face	1.0	0.847	0.740	0.348	**0.513**	0.277	Sad	–
Persevering Face	1.0	0.435	0.804	0.435	**0.804**	0.324	–	Disgust: A – Anger: G– Fear: S
Relieved Face	1.0	0.180	1.0	0.180	***0.409***	0.180	–	Calm: A & S– Fear: G
Smiling Face	1.0	0.677	0.365	0.677	*0.469*	**0.693**	–	Happy: A & S– Calm: G
Smiling Face with Smiling Eyes	1.0	0.847	0.703	**0.847**	0.703	0.595	Happy	–
Smirking Face	1.0	0.799	0.435	**1.0**	0.348	0.348	–	Love: A – Contempt: G– Calm: S
Unamused Face	1.0	0.705	0.348	**0.847**	0.348	0.277	Envy	–
Weary Face	1.0	0.659	0.584	**0.659**	**0.659**	0.257	–	Envy: A– Sad: G – Disgust: S
Winking Face	1.0	0.799	0.778	0.677	**0.778**	0.527	Love	–
Worried Face	1.0	0.513	0.738	0.129	**0.659**	0.374	–	Fear: A– Envy: G – Sad: S

*For columns labeled “Between older versions of each platform,” bold values represent the highest correlation coefficient between two platforms. For faces that were perceived as different emotions(s) among these platforms, the predicted highest correlation coefficient (based on shared perception) is in italics. For only the “relieved face” and the “disappointed face” was the prediction supported. A = Apple 9.1; G = Google Android 6.0; S = Samsung Wiz.*

### Analysis 1b: Correlation Across AUs for Faces Perceived as the Same Emotion vs. Different Emotion(s)

Twelve emoji faces were perceived as the same emotion across all three platforms. Twelve faces were perceived as a different emotion on one of the three platforms (i.e., two platforms shared a perceived emotion). Seven additional faces were perceived as a different emotion on *each of the three* platforms (see Table 2).

To test Hypothesis 1b, we compared the overall AU count and distribution of AUs across those emoji faces that were perceived as the same emotion (*n* = 35) vs. those perceived differently (*n* = 58) (on at least one other platform).

For the overall AU count, there was not a significant difference between the AUs for same- and differently-perceived emotions using a Mann Whitney *U*-test, *U*(1) = 0.011, *p* > 0.05. It is also worth noting that of the emoji faces perceived as the same emotion across platforms, 50.0% had a correlation among AUs exceeding 75%. The rate was barely less (45%) for emoji faces perceived as different emotions.

The distribution of AUs between same- and differently-perceived emotions was significantly different for three AUs, however: AU 46, *U*(1) = 928.0; AU 63, *U*(1) = 847.5; *crying, U(*1) = 789.0, *ps* < 0.05.

#### Summary

Overall, Hypothesis 1b was not supported: the distribution of AUs was not statistically different among faces perceived as the same emotion compared to faces perceived as a different emotion(s). Three AUs were significantly different between same- and differently-perceived emotions, however, suggesting that there are some AUs that might be helpful in distinguishing certain emotions from others (thus increasing the agreement of emotion category perception).

Note that of the 12 emoji faces which were perceived differently on one platform (but the same on the two others), only two faces showed relatively lower correlations among AUs compared to those perceived as the same emotion (see Table 5).

### Analysis Set 2a: Correspondence Between AUs for ICP Prototypes and Perceived Emotion

In our previous study, 228 English-speaking participants chose to which emotion category(ies) each of the 31 emojis belonged (Franco and Fugate, 2020). Participants randomly received all 31 emoji faces from either the Apple 9.1, Google Android 6.0, or Samsung Wiz platform. Emojis were shown individually for ten emotions (presented as words). Participants could indicate up to three emotion categories for each emoji face. Once an emotion category was selected, participants indicated the strength of that relationship on a 10-point Likert scale. Participants did not need to choose more than one emotion, but they needed to select at least one for each emoji face. For the purposes of this paper, we used the most frequent emotion category that participants indicated for each emoji face (for each of the three platforms). These results are also part of Table 2 in the Supplementary Files of that article Franco and Fugate (2020).

Table 6 presents the percentage of time each AU was used for each perceived emotion across platforms.

**TABLE 6:** Percentage of AUs (as a total of number of AUs) by Emotion.

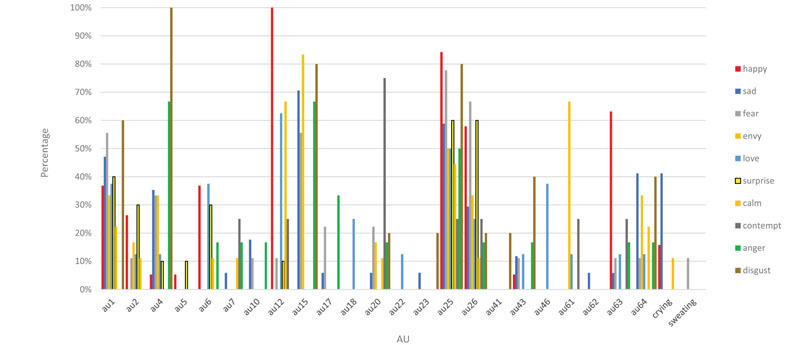

#### Across All Platforms and Versions

Of the 15 AUs identified for the ICP prototypes for the emotions we explored, we did not code for two: AU 9 and AU 54. Although we came up with a code for AU 14 and AU16, we never coded any instances of either. Therefore, we were able to compare codes on the 11 AUs common to the ICP prototypes and our emoji faces. We removed faces perceived as envy from these analyses, as there is no ICP prototype for envy.

To assess Hypothesis 2a, we used a Wilcoxon signed-ranks test to compare the distribution of AUs between the ICP prototype and our emoji faces. There was a significant difference using the Z transformation statistic, *Z*(87) = −5.15, *p* < 0.05 (mean rank ICP prototype AUs = 24.59, mean rank coded AUs = 39.32). Therefore, the distribution of AUs between the ICP prototypes overall and our coded AUs was different. Hypothesis 2a was not supported.

#### Individual Platforms

We next analyzed the distribution of these AUs by platform.

#### Apple

There was a significant difference between the distribution of AUs between the ICP prototype and our emoji faces, using the Z transformation statistic, *Z*(29) = −3.92, *p* < 0.05 (mean rank ICP prototype AUs = 5.0, mean rank coded AUs = 13.57). Thus, Hypothesis 2a was not supported on the Apple 9.1 platform.

#### Google Android

Between the ICP prototypes and our emoji faces on the Google Android platform, there was also a significant difference, *Z*(29) = −3.67, *p* < 0.05 (mean rank ICP prototype AUs = 8.13 and mean rank coded AUs = 14.48). Thus, Hypothesis 2a was not supported on the Google Android 6.0 platform.

#### Samsung

Lastly, between the ICP prototypes and our emoji faces on the Samsung platform, there was not a significant difference, *Z*(29) = −0.859, *p* > 0.05 (mean rank ICP prototype AUs = 10.17, mean rank coded AUs = 11.63). Therefore, only Samsung Wiz used AUs similarly to the ICP prototypes (across all emotions). Thus, overall, Hypothesis 2a was only supported for one platform.

### Prototype AUs by Emotion

To further explore the correlation and importance of AUs for each emotion prototype as it related to the perceived emotion, we next separated the results by emotion. The following numbers represent how many emoji faces were perceived as each emotion (across the three platforms): anger (*n* = 6), calm (*n* = 9), contempt (*n* = 4), disgust (*n* = 5), envy (*n* = 6), fear (*n* = 9), happy (*n* = 19), love (*n* = 8), sad (*n* = 17), and surprise (*n* = 10) (see Table 2).

#### Anger

Only one face was perceived across the three platforms as anger: “angry face.” Each platform had an additional face perceived as angry. The ICP prototype for anger is AU 4 and AU7 (see Table 1). A multinomial regression using AU 4 and AU 7 as predictors to obtain perceived emotion was significant, *X*^2^(18, *n* = 93) = 44.1, *p* = 0.001, Nagelkerke = .382 (McFadden = 0.108). AU 4 was a significant predictor of perceived emotion overall, *X*^2^(9, *n* = 93) = 34.8, *p* < 0.001, but not for anger. Only the absence of AU 4 predicted the emotion happy, *B* = 3.59 (SE = 1.37), Wald = 6.87, *p* < 0.01), and surprise, *B* = 2.90 (SE = 1.39), Wald = 4.35, *p* < 0.05. In fact, there were no classifications to anger using these two AUs (but see classification rate using all AUs, Hypothesis 2b below).

#### Calm

No faces were perceived as calm across the three platforms. Apple had two faces perceived as calm, Google Android had five faces, and Samsung had two faces. The ICP prototype for calm is AU 12 and AU 43 (see Table 1). A multinomial regression using AU 12 and AU 43 as predictors was significant, *X*^2^(18, *n* = 93) = 91.4, *p* < 0.001, Nagelkerke = 0.634 (McFadden = 0.225). AU 12 was a significant predictor of perceived emotion overall, *X*^2^(9, *n* = 93) = 81.45, *p* < 0.001, but not for calm. The absence of AU 12 significantly predicted surprise, *B* = 2.89 (SE = 1.27), Wald = 5.19, *p* < 0.05, and fear, *B* = 2.90 (SE = 1.33), Wald = 4.79, *p* < 0.05. There were no classifications to calm with these two AUs, however (but see classification rate using all AUs, Hypothesis 2b below).

#### Contempt

No faces were perceived as contempt across the three platforms. Apple and Google Android had one face perceived as contempt, and Samsung had two faces. The ICP prototype for contempt is AU 4, AU 14, and AU 25 (see Table 1). AU 14 was never coded. A multinomial regression using AU 4 and AU 25 as predictors was significant, *X*^2^(18, *n* = 93) = 46.73, *p* < 0.001, Nagelkerke = 0.400 (McFadden = 0.115). As mentioned before, AU 4 was a significant predictor of emotion. AU 25 was not a significant predictor or perceived emotion overall, although the presence of AU 25 significantly predicted happy, *B* = −2.726 (SE = 1.32), Wald = 4.29, *p* < 0.05. There were no classifications to contempt with these two AUs (but see classification rate using all AUs, Hypothesis 2b below).

#### Disgust

No faces were perceived as disgust across the three platforms. Apple, however, had two faces perceived as disgust; Google Android had two, and Samsung had one face perceived as disgust. The ICP prototype for disgust is AU 4, AU 6, AU 7, AU 9, AU 10, AU 25, and AU 26 (see Table 1). We did not have a code for AU 9. A multinomial regression using these six AUs as predictors was significant, *X*^2^(54, *n* = 93) = 108.57, *p* < 0.001, Nagelkerke = 0.698 (McFadden = 0.267). In addition to AU 4 and AU 12, which were previously identified as significant predictors, AU 6, AU 10, and AU 26 were also now identified as significant predictors overall, *X*^2^(9, *n* = 93) = 19.9, *p* < 0.05; *X*^2^(9, *n* = 93) = 17.45, *p* < 0.001; *X*^2^(9, *n* = 93) = 26.23, *p* < 0.01, respectively. None of the AUs significantly predicted any individual emotion, however, but the classification of disgust was 60% using these AUs (but see Hypothesis 2b).

#### Fear

No faces were perceived across the three platforms as fear, yet three faces on Apple, two on Google Android, and four on Samsung were perceived as fear. The ICP prototype for fear is AU 1, AU 2, AU 5, AU 7, and AU 25 (see Table 1). A multinomial regression using these five AUs as predictors was not significant, *X*^2^(45, *n* = 93) = 52.00, *p* > 0.05, Nagelkerke = 0.434 (McFadden = 0.128). AU 1 and AU 2 were marginally significant predictors of perceived emotion overall, however, *X*^2^(9, *n* = 93) = 16.58, *p* = 0.056, and *X*^2^(9, *n* = 93) = 16.59, *p* = 0.056, respectively. None of the AUs significantly predicted any individual emotion, and there were no correct classifications to fear (but see Hypothesis 2b).

#### Happy

Only three emoji faces were perceived as happy across all the three platforms: “face with tears of joy,” “smiling face with smiling eyes,” and “grinning face with smiling eyes.” An additional four emojis were perceived as happy on Apple, an additional two emoji faces on Google Android, and an additional four emoji faces on Samsung. The ICP prototype for happy is AU 6, AU 7, AU 12, AU 16, AU 25, and AU 26 (see Table 1). A multinomial regression using these six AUs as predictors was significant, *X*^2^(45, *n* = 93) = 134.65, *p* < 0.001, Nagelkerke = 0.775 (McFadden = 0.331). AU 6, AU 12, and AU 26 were identified as significant predictors (as previously mentioned), although none of them predicted any individual emotion. Despite this, these AUs classified happiness 94.7% (the same as when all 11 coded AUs were added to the model, see Hypothesis 2b, below).

#### Love

Two faces were perceived across the three platforms as love: “winking face” and “kissing face.” Both Apple and Google Android had one additional face perceived as love. The ICP prototype for love is AU 6, AU 7, AU 12, and AU 25 (see Table 1). A multinomial regression using these four AUs as predictors was significant, *X*^2^(36, *n* = 93) = 116.87, *p* < 0.001, Nagelkerke = 0.725 (McFadden = 0.288). AU 6 and AU 12 had been previously identified as significant predictors, and maintained here. The absence of AU 12 predicted fear, *B* = 3.11 (SE = 1.42), Wald = 4.82, *p* < 0.05), and surprise, *B* = 3.03, (SE = 1.37), Wald = 4.93, *p* < 0.05. Zero percent of faces were classified to love (but see Hypothesis 2b).

#### Sad

Three faces were perceived as sad across the three platforms: “loudly crying face,” “pensive face,” and “crying face.” Apple, however, had two additional faces perceived as sad, whereas Google Android and Samsung had an additional three faces each perceived as sad. The ICP prototype for sad is AU 4, AU 43, and AU 54 (see Table 1). We did not code for AU 54. A multinomial regression using these two AUs as predictors was significant, *X*^2^(18, *n* = 93) = 45.03, *p* < 0.001, Nagelkerke = 0.389 (McFadden = 0.111). As previously indicated, AU 4 was a significant predictor of perceived emotion overall. The absence AU 4 significantly predicted happy, *B* = 2.35, (SE = 1.15), Wald = 4.19, *p* < 0.05. These AUs predicted sadness 47.1% (which was substantially lower than when all 11 AUs were included, see Hypothesis 2b).

#### Surprise

Two faces were perceived as surprise across the three platforms: “face without mouth” and “face with open mouth.” Apple had two additional faces perceived as surprise, whereas Google Android and Samsung had one additional face each. The ICP prototype for surprise is AU 1, AU 2, AU 5, and AU 25 (see Table 1). A multinomial regression using these four AUs as predictors was not significant, *X*^2^(36, *n* = 93) = 44.60, *p* > 0.05, Nagelkerke = 0.386 (McFadden = 0.110). AU 2 was a significant predictor overall (as previously indicated). These AUs classified surprise 20% (substantially lower than with all 11 AUs, see Hypothesis 2b).

#### Summary

To summarize, AUs that were significant predictors overall of an emotion category (although not specifically which one) included AU 1, AU 2, AU 4, AU 6, AU 10, AU 12, and AU 26. None of the 11 AUs represented in the ICP prototypes for the emotions we studied predicted any one emotion category specifically, except AU 25 which predicted happy. Interestingly, AU 25 is part of the ICP prototype for *all but* three of the emotions we studied, yet we only found that its presence predicted happy. Of the other AUs, only AU 10 is thought to be specific (disgust)^7^.

### Analysis 2b: Perceived Emotion Classification

To test Hypothesis 2b, we used a multinomial logistic regression to test whether the 11 AUs from the ICP prototypes could better predict the perceived emotion category across emoji faces. We also compared individual platforms/versions.

#### Across Platforms

We first computed the MLR on the 11 shared AUs across platforms for all ten emotions. The dependent variable was the perceived emotion category. The model produced was significant, *X*^2^(99, *n* = 93) = 226.261, *p* < 0.001, Nagelkerke = 0.924 (McFadden = 0.557). Likelihood ratio tests were significant for seven AUs: AU 1 (*X*^2^(9) = 42.63, *p* < 0.001); AU 4 (*X*^2^(9) = 45.55, *p* < 0.001); AU 6 (*X*^2^(9) = 21.10, *p* < 0.05); AU 7 (*X*^2^(9) = 21.90, *p* < 0.05); AU 12 (*X*^2^(9) = 57.84, *p* < 0.001); AU 25 (*X*^2^(9) = 17.67, *p* < 0.05); AU 26 (*X*^2^(9) = 29.38, *p* = 0.001). None of the individual emotions were significantly predicted, however, with these AUs.

The overall classification rate of emotions to their predicted category was 58.1%. Table 7 shows the classification matrix. Overall, *happy* was the best classified at 94.7% (*n* = 19). One incorrect classification was assigned to calm. *Sad* had the next best classification rate at 88.2% (*n* = 17). One incorrect classification went to fear. *Anger* had a classification rate of 83.3% (*n* = 6), with incorrect classification assigned to disgust. *Disgust* had a classification rate of 80% (*n* = 5), with one incorrect classification assigned to sad. *Surprise* had a 60.0% classification rate (*n* = 9). Incorrect classifications were mainly assigned to sad, followed by one each to fear and to happy. *Fear* had a 33.3% classification rate (*n* = 9): Fear was misclassified mainly as sad and surprise, followed by a tie between calm and envy. C*alm* had a classification rate of 22.2% (*n* = 9). Incorrect classifications were mainly assigned to happy, followed by a tie between surprise, anger, and sad. *Envy* had a poor classification rate at 16.7% (*n* = 6). Incorrect classifications were mainly assigned to sad, followed by fear, love, and disgust. *Love* also had a poor classification rate at 12.5% (*n* = 8): Love was misclassified as calm, followed by happy, and then sad and surprise. *Contempt* had the worst classification rate (0.0%, *n* = 4), with incorrect classifications split among sad, surprise, calm, and anger. These results are generally in line with classification rates of AUs to human emotion categories. Specifically, individual instances of faces perceived as happy, anger, and fear contain more of the prototypical AUs, compared to contempt, sadness, and disgust, which generally show less overlap with the proposed codes (Cordaro et al., 2018).

**TABLE 7:** Classification Matrix using all 11 AUs common to ICP prototypes for Studied Emotions.

	**Happy**	**Sad**	**Fear**	**Envy**	**Love**	**Surprise**	**Calm**	**Contempt**	**Anger**	**Disgust**
Happy	94.7	0.0	0.0	0.0	0.0	0.0	5.3	0.0	0.0	0.0
Sad	0.0	88.2	11.8	0	0.0	0	0	0	0	0
Fear	0.0	22.2	33.3	11.1	0.0	22.2	11.1	0.0	0.0	0
Envy	0.0	33.3	16.7	16.7	16.7	0	0.0	0.0	0	16.7
Love	25.0	12.5	0.0	0	12.5	12.5	37.5	0.0	0.0	0.0
Surprise	10.0	20.0	10.0	0	0.0	60.0	0.0	0.0	0.0	0.0
Calm	44.4	11.1	0.0	0	0.0	11.1	22.2	0	11.1	0.0
Contempt	0.0	25.0	0.0	0	0.0	25.0	25.0	0	25.0	0.0
Anger	0.0	0.0	0.0	0.0	0	0.0	0.0	0	83.3	16.7
Disgust	0.0	20.0	0.0	0.0	0.0	0.0	0.0	0.0	0	80.0

#### Individual Platforms

We next compared the three platforms. Apple and Samsung both produced marginally significant models: Apple, *X*^2^(90) = 112.83, *p* = 0.052, Nagelkerke = 0.987 (McFadden = 0.841); Samsung, *X*^2^(90) = 112.58, *p* = 0.054, Nagelkerke = 0.987 (McFadden = 0.849). The model for Google Android was not significant, *X*^2^(81) = 196.76, *p* > 0.05, Nagelkerke = 0.968 (McFadden = 0.717). Interesting, however, when comparing the AIC values, the best fit was Google Android. This is likely because there were fewer AUs coded for Google Android, but of those, there was slightly better classification: Google Android AIC = 196.92, followed by Apple AIC = 208.05, and AIC Samsung = 210.87. The lower the value, the “better” fit of the model.

Yet, Samsung had the highest overall classification rates, with 83.9% (Samsung: range = 0% disgust to 100% for happy, fear, envy, surprise, and anger). Apple had an overall classification rate of 80.6% (range: 0% for calm and contempt to 100% for fear, envy, love, anger, and disgust). By comparison, Google Android had a correct classification rate of 64.5% (range 0% for fear and contempt to 100% for sad).

#### Summary

To summarize, 11 AUs were better at predicting perceived emotions than only the ones in the ICP prototype for each emotion. There were differences in how well each platform classified individual emotions from AUs. For example, Google Android only had two faces perceived as fear but did not classify either correctly, whereas Apple and Samsung had three and four faces perceived as fear and classified them all correctly. Apple did a poor job classifying calm (0%) (*n* = 2), but classification was 50% (*n* = 2) on Samsung and 60% (*n* = 5) on Google Android. Finally, Samsung did a poor job classifying disgust (0%) (*n* = 1), but Google Android had a 50% classification rate (*n* = 2) and Apple had a 100% classification rate (*n* = 2).

## Discussion

In this manuscript, we created an adapted emoji-FACS system to explore whether emoji faces (from an anatomical perspective) look similar across platforms/versions, and whether the anatomical configurations are shared with human expressions. Although FACS was not designed for nonhuman faces, it has been adapted and validated for a number of species over the years (e.g., chimpFACS, Parr et al., 2007; and MaqFACS, Parr et al., 2010). Clearly emoji faces are not human (or nonhuman faces), but they are perceived as faces with emotional content.

Once we established the emoji-FACS rubric, the first goal was to systematically compare AUs for emoji faces across platforms and versions. Although emoji faces were designed for the purpose of communicating emotional information, there is little agreement about what specific emotion an individual face is perceived as. We found that different platforms and versions not only often relied on different AUs, but also often that the frequency of AUs was different across platforms and versions (Hypothesis 1a). In addition, faces perceived as the same emotion and those perceived as different emotion(s) were equally diverse in their use and distribution of AUs (Hypothesis 1b). In a few instances, certain AU counts did differ between faces perceived as the same vs. different emotion(s), but this could be attributed to the fact that these AUs were only present in one emotion category and had good predictive validity (e.g., tears for sadness and winks for love).

The second goal was to assess whether emoji-coded AUs were similar to the AUs in the ICP prototypes for the same perceived emotion. Across platforms and versions, we found that AUs common to emotion prototypes were used in emoji faces, but AUs did not predict *specific* emotion categories (Hypothesis 2a). Similar results were found when we included all the AUs in our model to predict emotion category, although overall classification rates increased when we did so. Our model was moderately good at predicting emotion: The average across categories was 58.1%. Specifically, happy, sad, anger and disgust were best predicted overall, but there were substantial differences among platforms in the individual emotion classification rates (Hypothesis 2b). Google Android showed the least predictive ability, yet it produced the best fitting model of the three platforms. This was likely because it used fewer AUs, but used them in more consistent ways. None of the AUs predicted a specific emotion category, however, except AU 25. Rather than outright predicting a specific emotion category, individual AUs seemed to narrow down to what emotion category an emoji face might belong by knowing *what category it is not.* Thus, the majority of AUs only give some predictive validity.

Although we did not test a model which included all 26 of our codable AUs as predictors of emotion category (rather than the 11 AUs shared with ICP prototypes), there is little doubt that some of these additional AUs would have been significant predictors (e.g., crying was only used in faces perceived as sad, and AU 18, AU 22, and AU 46 were only used in faces perceived as love). Thus, it is reasonable to assume that some AUs we coded (even though not part of the ICP prototypes for human facial expressions) are specific to an emotion category.

This finding is consistent with the results of a recent study using emoji-like faces (Betz et al., 2019). In that study, participants were asked to which emotion category each face belonged. Faces were either presented in the context of emotion words or not. Overall, adding emotion words increased emotion agreement for these faces, as adding emotion words increases the agreement among raters for human facial depictions of emotion (for reviews, see Lindquist and Gendron, 2013; Lindquist et al., 2016; Barrett et al., 2019). Yet, some emojis in that study were less affected by the context of words. For instance, people largely agreed (without any context) that the face with wide eyes and a gaping mouth was surprise, even without the added context of emotion words.

The Theory of Constructed Emotion (Barrett, 2017; Barrett et al., 2019) suggests that the human brain is constantly predicting what a stimulus is (e.g., a face) and to what emotion category it might belong (e.g., anger or fear). It recognizes that emotion perception (and the perception of categories of the mind, more generally) is the product of such predictions. According to this view, people perform a type of “affective calculus” in which their brain is constantly predicting (based on provided labels, situational context, and previous knowledge) what a stimulus is and to what category it belongs (see also Betz et al., 2019). Of course, predictions are built (at least initially) on information from the world- in the case of emotion perception, from the information our body senses either within ourselves or other people. Some of these changes can become associated with emotional meaning when occurring in a specific context. Perhaps then we can best think of emoji faces (much like human faces) as providing a starting point for more refined predictions. Faces, like voices, bodily postures, and the like aren’t diagnostic of emotions, but they can help to narrow the outcome of our brain’s predictions. We might then think of this core set of AUs (plus perhaps a few other which might be specific to emoji faces) as helping to narrow which emotion category a face belongs. This idea seems particularly in line with our findings that the core AUs did not predict a specific emotion well (much in the same way AUs do not predict specific emotions from a human face very well), but they contributed to the process. Although we did not test this theory specifically, in future studies adding a context (whether verbal or pictorial) should facilitate perception and therefore increase agreement among raters as to which emotion category an emoji face belongs.

These findings are also consistent with Channel Expansion Theory in Communication (Carlson and Zmud, 1999), in which exposure to electronic communication enhances a receiver’s knowledge about those platforms and thus refines possible interpretations. Indeed, receivers develop their computer-mediated communication skills through experience with others using the same medium and the feedback they receive from others. Therefore, experience with online communication (in which emoji faces are used) allows receivers to develop and ultimately better convey information, such as that about emotion (Gudykunst, 1997).

### Implications

So, what does this mean for computer programmers in charge of the physical renderings of emoji faces? Two things jump to mind. The first is that programmers should be aware of just how different “equivalent” emojis really are in terms of their appearance. They must also be aware that, more often than not, “equivalent” emoji faces not only look different, but are also perceived as different emotion categories.

Second, emoji faces do not appear in isolation. Although a single emoji can be sent or texted to an individual, it is in reference to something either explicitly communicated or implicitly understood between the two parties. Therefore, regardless of the individual theory of emotion to which a person ascribes, there is likely interplay between a face and the context (e.g., Trope, 1986; Aviezer et al., 2008, 2011). Future work should therefore also consider the usage of emojis in context and elaborate on how the context can affect emotional interpretation (see Walther and D’Addario, 2001; Kelly, 2015).

### Limitations and Future Directions

This research has several limitations. The first is the selection of possible choices (and number) of emotions and emojis. This study used 31 emojis (depicted on three electronic platforms). The Unicode system now has 3,136 emoji characters, 92 of which are emoji faces (Unicode, 2020). We also only included three major platforms, and there are many others, including Facebook, Twitter, and WhatsApp. In addition, we only investigated ten emotions, and as noted, other researchers have proposed more (Cordaro et al., 2018).

Another limitation is that we only adapted 26 codes from the FACS system, which includes more than 65 (with head and eye positions). While ICP prototypes, however, only use a subset (15 AUs, not including body postures which are sometimes included), our codes included only 11 of these 15. One way that we tried to address this was by including additional AUs. This included things like eye gaze, lip puckers and funnelers, *crying*, and including other potential “candidates” for specific emotion AUs (e.g., AU 20 and AU 15).

Perhaps the largest limitation is that we used only the ICP prototypes from Westerns, and such configurations do not likely apply to displays from Eastern countries (Cordaro et al., 2018). For example, East Asian models show less distinction between emotions (see also Jack et al., 2012). Related to this limitation, our perceived emotions from emoji faces came from English-speakers who all resided in the United States and were mainly between 18 and 24 years old (see Franco and Fugate, 2020).

We recommend that future empirical research on emojis both broadens the repertoire of emojis (also opens up to additional platforms) and also considers the perceived emotion given from non-Western individuals. Ultimately, however, it is in the hands of the programmers to decide how to translate an emoji in newer versions across platforms. That said, we strongly advocate that programmers also consider the role that emoji labels play (e.g., “confused” face, “disappointed” face) as they might be in opposition to the perceived emotion. Moreover, we strongly advocate that the field of emotion, and general nonverbal communication as a whole, explore the role that the perceiver’s conceptual knowledge and that situational cues play in interpreting the rudimentary structural information that exists in the face.

## Data Availability Statement

The datasets presented in this study can be found in online repositories. The names of the repository/repositories and accession number(s) can be found below: Emoji Faces and Coding at OSFHome: https://osf.io/9dzfw/.

## Ethics Statement

The studies involving human participants were reviewed and approved by the University of MA - Dartmouth IRB # 16.052. A. Kareberg, Office of Institutional Compliance. The ethics committee waived the requirement of written informed consent for participation.

## Author Contributions

JF performed all coding and analyses. Both authors conceived of the idea and wrote the manuscript.

## Conflict of Interest

The authors declare that the research was conducted in the absence of any commercial or financial relationships that could be construed as a potential conflict of interest.

## Footnotes

1 We thank a reviewer for pointing this out.

2 Envy does not have an ICP.

3 face-and-emotion.com/dataface/facs/guide/FACSIV1.html

4 AU 54 is one of the ICP prototype AUs.

5 Only a small percentage (less than 5%) of total codes contained R/L.

6 Indicated with cheek blushing, no cheeks otherwise.

7 AU 15 is not included in the ICP for sadness, despite all previous studies noted in Table 1. AU 20 is also not included for fear, despite all previous studies noted in the Table 1.
